# Palmitoylation of the β4-Subunit Regulates Surface Expression of Large Conductance Calcium-activated Potassium Channel Splice Variants*

**DOI:** 10.1074/jbc.M113.461830

**Published:** 2013-03-16

**Authors:** Lie Chen, Danlei Bi, Lijun Tian, Heather McClafferty, Franziska Steeb, Peter Ruth, Hans Guenther Knaus, Michael J. Shipston

**Affiliations:** From the ‡ Centre for Integrative Physiology, College of Medicine and Veterinary Medicine, University of Edinburgh, Edinburgh EH8 9XD, Scotland, United Kingdom,; the § Division of Molecular and Cellular Pharmacology, Innsbruck Medical University, A-6020 Innsbruck, Austria, and; the ¶ Division of Pharmacology and Toxicology, Institute of Pharmacy, University of Tuebingen, 72076 Tuebingen, Germany

**Keywords:** Ion Channels, Membrane Transport, Potassium Channels, Protein Palmitoylation, Signal Transduction, S-Acylation

## Abstract

Regulatory β-subunits of large conductance calcium- and voltage-activated potassium (BK) channels play an important role in generating functional diversity and control of cell surface expression of the pore forming α-subunits. However, in contrast to α-subunits, the role of reversible post-translational modification of intracellular residues on β-subunit function is largely unknown. Here we demonstrate that the human β4-subunit is *S*-acylated (palmitoylated) on a juxtamembrane cysteine residue (Cys-193) in the intracellular C terminus of the regulatory β-subunit. β4-Subunit palmitoylation is important for cell surface expression and endoplasmic reticulum (ER) exit of the β4-subunit alone. Importantly, palmitoylated β4-subunits promote the ER exit and surface expression of the pore-forming α-subunit, whereas β4-subunits that cannot be palmitoylated do not increase ER exit or surface expression of α-subunits. Strikingly, however, this palmitoylation- and β4-dependent enhancement of α-subunit surface expression was only observed in α-subunits that contain a putative trafficking motif (… REVEDEC) at the very C terminus of the α-subunit. Engineering this trafficking motif to other C-terminal α-subunit splice variants results in α-subunits with reduced surface expression that can be rescued by palmitoylated, but not depalmitoylated, β4-subunits. Our data reveal a novel mechanism by which palmitoylated β4-subunit controls surface expression of BK channels through masking of a trafficking motif in the C terminus of the α-subunit. As palmitoylation is dynamic, this mechanism would allow precise control of specific splice variants to the cell surface. Our data provide new insights into how complex interplay between the repertoire of post-transcriptional and post-translational mechanisms controls cell surface expression of BK channels.

## Teaser

**Background:** The role of post-translational modification of regulatory β-subunits in the control of large conductance potassium (BK) channels is largely unknown.

**Results:** β4-subunit palmitoylation controls surface trafficking of BK channel α-subunit splice variants.

**Conclusion:** Palmitoylation of β4 masks an α-subunit trafficking motif to control surface delivery.

**Significance:** Palmitoylation of regulatory subunits provides a dynamic mechanism to control surface trafficking of specific BK channel variants.

## Introduction

The pore-forming α-subunits of large conductance voltage- and calcium-activated potassium (BK) channels assemble with a number of accessory regulatory β- and γ-subunits (1, 2). These regulatory subunits provide a mechanism to increase the functional diversity of BK channels in different tissues by modifying their calcium and/or voltage sensitivity, channel kinetics, surface expression, or regulation by a range of signaling molecules and toxins. Indeed, loss of function of these regulatory subunits is associated with disruption of normal physiological processes ranging from control of vascular tone (3) to excretion of potassium from the kidney (4, 5) and neuronal excitability (6).

Thus, mechanisms that dynamically control the functional regulation of α-subunits by regulatory subunits represent important determinants of physiological control. Indeed, BK channels are dynamically regulated by a diverse range of reversible post-translational modifications. However, in contrast to the extensive post-translational modification of intracellular residues of the pore-forming α-subunit, reversible post-translational modification of regulatory subunits is very poorly characterized.

Increasing evidence supports an important role for the only reversible lipid post-translational modification of proteins, *S*-acylation (palmitoylation), as an important mechanism to control a wide diversity of ion channels, including BK channels (7). Here we demonstrate that the BK channel regulatory β4-subunit is *S*-acylated (palmitoylated) at a cysteine residue in the C terminus juxtaposed to the second transmembrane domain. Palmitoylation of the β4-subunit controls surface expression of BK channels and thus represents an important additional regulatory step in controlling BK channel properties and function.

## EXPERIMENTAL PROCEDURES

### Expression Constructs

Full-length BK channel ZERO α-subunit splice variants (coding sequence starts and ends in amino acids MDA … DEC, respectively, also referred to as MDA-DEC (see Fig. 4*A*)) with either an N-terminal FLAG tag (FLAG-ZERO) or an N-terminal FLAG and C-terminal HA tag (FLAG-ZERO-HA) in pcDNA3.1 were described previously (8). To generate FLAG-tagged splice variants differing in the N or C termini, a construct with coding sequence starting and ending MAN … ERL (generous gift of Dr. Jon Lippiat, University of Leeds (9)) was first subcloned into pcDNA3.1 using NheI and NotI. An N-terminal FLAG tag was generated by PCR amplification using forward and reverse primers, forward 5′-ACGGTACCATGGATTACAAGGATGACGACGATAAGGCAAATGGTGGC-3′, and reverse 5′-CACTATCATGAGCTCAAACAC-3′, to add the FLAG tag with a KpnI digestion site upstream of its start codon. Amplicons were TOPO-cloned using pCRII-TOPO (Invitrogen) and then subcloned into the MAN-ERL backbone in pcDNA3.1 using KpnI and PpuMI. To generate the MDA-ERL variant, an N-terminal KpnI and PpuMI fragment from FLAG-ZERO (MDA-DEC) was subcloned into the MAN-ERL backbone. To engineer the C-terminal heptapeptide REVEDEC from the ZERO (MDA-DEC) variant into the MAN-ERL variant to generate MAN-REVEDEC, the last 7 residues of MAN-ERL were swapped with REVEDEC using PCR primers, forward 5′-GTC CTT CCC TAC TGT TTG-3′, and reverse 5′-CCTCTAGATCAACATTCATCTTCAACTTCTCTCTTCTGTTTGTCCCGGG-3′, and the subsequent amplicon was ligated into a PacI and XbaI backbone from MAN-ERL.

To generate site-directed mutants and epitope-tagged constructs of the β4-subunit, a human construct (generous gift of Dr. Jon Lippiat, University of Leeds (9)) was used as template and subcloned into pcDNA3.1. The palmitoylation-deficient mutant C193A was generated using: forward 5′-TGTGGTCCTGACCATCGCTGCCAAGAGCTTGGCG-3′, and reverse 5′-ACCGCCAAGCTCTTGGCAGCGATGGTCAGGACCAC-3′ primers. The trafficking-competent mutant (KAA*X*), in which Lys-206 and Arg-207 were mutated to alanine, was generated by PCR amplification using a forward internal CMV primer within the vector backbone and a reverse primer 5′-ACGGGCCCTCTAGATTAAGAGAACTTGGCCGCCTTC-3′ and an NheI and XbaI fragment ligated into the β4-subunit backbone. Constructs with a C-terminal Myc tag (β4-Myc_c_) were also trafficking-competent and generated by PCR using forward 5′-ACTGCTTACTGGCTTATCG-3′, and reverse 5′-ACTCGAGTTAAGATCCTCTTCTGAGATGAGTTTTTGTTCAGAGAACTTGC-3′ primers, TOPO-cloned, digested with NheI and XbaI, and ligated into pcDNA3.1. To generate β4-subunits with an extracellular Myc tag (β4-Myc_e_), the KOD mutagenesis kit (Novagen) using primer pairs forward 5′-TGGAAAGATGAGCAGAAACTCATCTCAGAAGAGGATCTTGGTTCCCAGCC-3′, and reverse 5′-TGGCTGGGAACCAAGATCCTCTTCTGAGATGAGTTTCTGCTCATCTTTCC-3′ was used on the respective constructs. Inclusion of the Myc_e_ tag had no effect on wild-type (WT) or C193A mutant channel expression or localization. All constructs were verified by sequencing.

### Cell Culture, Transfection, and Imaging

HEK293 cells and N2a neurons were maintained in DMEM with 10% FCS. For imaging experiments, cells were plated on poly-d-lysine-coated glass in 6-well cluster plates at 15–20% confluency, and 24 h later, they were transfected with the respective plasmids using ExGen 500 and used 48 h after transfection. For N2a, cells were differentiated for 48 h after transfection in DMEM containing 1% BSA.

Quantitative cell surface labeling of N-terminal FLAG epitope-tagged BK channel α-subunits in nonpermeabilized cells was performed using mouse monoclonal anti-FLAG M2 antibody (Sigma, 50 μg/μl) and secondary anti-mouse Alexa Fluor 543 (Invitrogen, 1:1000). Cells were then fixed in 4% paraformaldehyde for 30 min, permeabilized with 3% Triton X-100 for 10 min, and blocked with phosphate-buffered saline containing 3% bovine serum albumin plus 0.05% Tween 20 for 1 h. For total BK channel expression, either the intracellular C-terminal HA epitope tag was probed with anti-HA polyclonal rabbit antibody (Zymed Laboratories Inc. 1:500) followed by Alexa Fluor 647 (Molecular Probes, 1:1000) or the FLAG tag was probed with anti-FLAG antibody with anti-mouse Alexa Fluor 488 (1:1000). To detect β4-subunits, two approaches were used. For β4-subunits lacking an epitope tag, we used a mouse monoclonal antibody targeted to an extracellular epitope of β4 (NeuroMab clone L18A/3). In nonpermeabilized and permeabilized conditions, primary antibody dilutions were 1:300 and 1:1200, respectively, with anti-mouse secondary Alexa Fluor 488 or Alexa Fluor 543. For β4-subunits with a Myc epitope tag, the extracellular Myc_e_ tag was detected using rabbit anti-Myc (Immune Systems) at 1:300 prior and anti-rabbit secondary antibody conjugated to either Alexa Fluor 488 or Alexa Fluor 647 prior to fixation and permeabilization. Total β4-subunit expression (Myc_c_) was determined following cell fixation and permeabilization as above by probing with rabbit anti-Myc (Immune Systems) at 1:1000 and anti-rabbit secondary antibody conjugated to either Alexa Fluor 488 or Alexa Fluor 647 (1:1000) as appropriate. Cells were mounted in Mowiol and dried at room temperature in the dark overnight before image acquisition.

Confocal images were acquired on a Zeiss LSM510 laser scanning microscope, using a 63× oil Plan Apochromat (NA = 1.4) objective lens, at Nyquist sampling rates in multitracking mode to minimize channel crosstalk. Three-dimensional image stacks were deconvolved using Huygens (Scientific Volume Imaging), and cell surface expression of full-length channels was determined by quantitative immunofluorescence by calculating the surface (FLAG) to total channel protein (−HA or intracellular FLAG) ratio using ImageJ (National Institutes of Health). For co-localization experiments with endoplasmic reticulum (ER),^2^ co-localization was assayed by co-transfection of the channel subunits with pdsRed-ER (Clontech). Confocal images were acquired and deconvolved as above, and Pearson's correlation coefficient (*R*) was determined using ImageJ (National Institutes of Health) with an *R* value of +1 indicating 100% co-localization.

### Palmitoylation Assays and Western Blotting

#### 

##### CSS-Palm Prediction

We exploited the published web-based CSS-Palm palmitoylation algorithm v3.0 (10) to predict cysteine residues within the entire coding sequence of the murine and human β4-subunits with prediction set to the highest cut off.

##### [^3^H]Palmitic Acid Incorporation

Transfected HEK293 cells were incubated in DMEM containing 10 mg/ml fatty acid free BSA for 30 min at 37 °C before incubation with 0.25 mCi/ml [^3^H]palmitic acid (PerkinElmer Life Sciences) for 4 h at 37 °C essentially as described (11, 12). Cells were lysed in 150 mm NaCl, 50 mm Tris-Cl, 1% Triton X-100, pH 8.0, and centrifuged, and channel fusion proteins were captured using magnetic microbeads (μMACS^TM^ epitope tag isolation kits, Miltenyi Biotech). Following extensive washing, captured proteins were eluted, separated by SDS-PAGE, transferred to nitrocellulose membranes, dried, and exposed to light-sensitive film at −80 °C using a KODAK BioMax TranScreen LE (Amersham Biosciences). The same membrane was then reprobed with either an anti-β4 antibody (NeuroMab L18A/3) or an anti-Myc tag as appropriate.

##### Acyl-RAC of Mouse Cerebellum

Acyl-RAC of mouse cerebellum was performed with a modification of the acyl-RAC method described by Forrester *et al.* (13). Briefly, cerebellar from mice aged 8–12 weeks were rapidly isolated and immediately homogenized with a Dounce on ice in lysis buffer containing 25 mm NaCl, 25 mm HEPES, 1 mm EDTA at pH 7.5 containing a protease inhibitor mixture and further disrupted through a 25-gauge needle. Lysates were centrifuged for 5 min at 3,000 rpm, and the supernatant centrifuged at 20,000 × *g* for 30 min with the pellet resuspended in lysis buffer containing 0.5% Triton X-100. Protein was diluted to 2 mg/ml in blocking buffer (100 mm HEPES, 1 mm EDTA, 2.5% EDTA, pH 7.5), and free thiols blocked with 0.1% methyl methanethiosulfonate at 40 °C for 4 h. Proteins were precipitated with ice-cold acetone, the pellet was washed five times with 70% acetone, and the final pellet was resuspended in binding buffer (100 mm HEPES, 1 mm EDTA, 1% SDS, pH 7.5). Half of the resuspension was incubated with 250 mm HEPES or 250 mm neutral hydroxylamine, and proteins were captured on thiopropyl-Sepharose beads for 2.5 h at room temperature. Beads were washed five times in binding buffer, and proteins were eluted in elution buffer containing 100 mm HEPES, 1 mm EDTA, 1% SDS, 50 mm DTT, pH 7.5. Eluates were subject to SDS-PAGE, transferred to PVDF, and probed with anti-β4 antibody as above.

### Electrophysiology

Macropatch recordings were performed using the inside-out patch clamp configuration at room temperature essentially as described (14). Briefly, the extracellular recording solution was composed of 140 mm KMeSO_3_, 2 mm KCl, 20 mm HEPES, 2 mm MgCl_2_, pH 7.3. The internal solution was composed of 140 mm KMeSO3, 2 mm KCl, 20 mm HEPES, 5 mm HEDTA, pH 7.3, with CaCl_2_ added to give a free Ca^2+^ concentration of 10 μm. Voltage protocols and acquisition were controlled using an Axopatch 200B amplifier and Digidata 1440A using pCLAMP10. Conductance-voltage (*G*/*V*) relationships were constructed from tail currents recorded using test pulses from −100 to 120 mV followed by a step to a negative voltage (−80 mV), and *V*_0.5 max_ was determined from Boltzmann fits of the normalized *G*/*V* curves. Activation and deactivation time constants were determined by fitting to an exponential function.

### Statistical Analysis

All data are presented as means ± S.E. with *N* = number of independent experiments and *n* = number of individual cells analyzed in imaging assays. Data were analyzed by ANOVA with post hoc Dunnett's test with significance set at *p* < 0.05.

## RESULTS

### 

#### 

##### β4 Is Palmitoylated at a Juxtamembrane C-terminal Cysteine Residue

**FIGURE 1.**
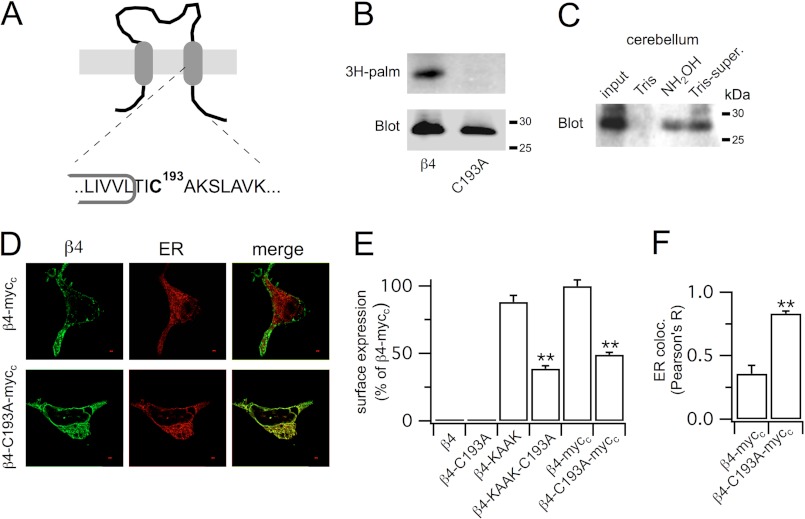
**Palmitoylation controls exit of the β4-subunit from the endoplasmic reticulum.** *A*, schematic of the β4 regulatory subunit of large conductance calcium- and voltage-activated potassium (BK) channels indicating the palmitoylated cysteine residue (Cys-193) juxtaposed to the intracellular C terminus of the second transmembrane domain. *B* representative fluorographs of [^3^H]palmitate (*3H-palm*) incorporation and corresponding Western blot (anti-Myc) of the wild-type β4-subunit and the alanine mutant C193A. *C*, acyl-RAC of murine cerebellum with Western blot probed with anti-b4. *D*, representative single confocal images of the β4 and C193A mutant expressed in HEK293 cells and co-labeled for the ER. *Scale bars* are 2 μm. *E* and *F*, bar graphs of membrane expression (expressed as a percentage of wild-type β4) (*E*) and co-localization with the ER (expressed as Pearson's correlation coefficient, *R*) (*F*) of the wild-type β4 and C193A mutant. Data are means ± S.E. *N* >5, *n* >200. **, *p* < 0.01 when compared with wild-type β4 group, ANOVA with post hoc Dunnett's test.

Using the CSS-Palm v3.0 palmitoylation prediction algorithm at high threshold (10), we identified a single predicted cysteine residue (Cys-193, CSS-Palm prediction score = 1.67; Fig. 1*A*), highly conserved across species, that is juxtaposed to transmembrane domain 2 (TM2) in the intracellular C terminus of the human β4-subunit (gene name *KCNMB4*). To test whether β4-subunits are palmitoylated, we took two approaches. Firstly, the β4-subunit was transiently expressed in HEK293 cells that were metabolically labeled with [^3^H]palmitate. Immunoprecipitation of the β4-subunits revealed robust incorporation of [^3^H]palmitate into β4-subunits (Fig. 1*B*). Mutation of the predicted palmitoylated cysteine residue Cys-193 to alanine (C193A) abolished [^3^H]palmitate incorporation without affecting protein expression of the mutated C193A β4-subunit (Fig. 1*B*). Secondly, using acyl-RAC (13) that allows determination of hydroxylamine-sensitive thioester bonds that couple *S*-acylated cysteine residues to their cognate lipid, we identified β4-subunit *S*-acylation in native mouse brain (Fig. 1*C*).

##### β4-Subunit Palmitoylation Controls Surface Expression and ER Exit

In many proteins, *S*-acylation controls trafficking and surface delivery of transmembrane proteins. To examine whether palmitoylation of β4-subunits affects their surface expression and trafficking *per se*, we undertook quantitative immunofluorescence assays. Using an antibody that recognizes an extracellular epitope expression of the WT β4-subunits in HEK cells revealed no significant surface expression (Fig. 1*E*) and predominant intracellular retention in the ER in agreement with previous studies (15). The C193A palmitoylation-deficient mutant had no significant effect on β4-subunit expression or localization (Fig. 1*E*). To improve the sensitivity of β4-subunit detection at the cell surface expression, we also engineered a β4-subunit with a Myc tag (Myc_e_) in the extracellular domain between transmembrane domains 1 and 2. Probing for the Myc_e_ tag revealed low, but detectable, levels of β4-subunit surface expression, with predominant intracellular ER retention, and surface expression was abolished below the limit of detection with the C193A mutant.

β4-Subunits are retained in the ER by a putative ER retention signal (KK*XX*) in the C terminus of the subunit (15). Thus, to improve the signal-to-noise ratio of our assay, we engineered two trafficking-*competent* β4-subunits to allow characterization of the role of palmitoylation in β4-subunit trafficking. Firstly, we mutated the central Lys-206 and Arg-207 amino acids of the KK*XX* ER retention motif to alanine (KAA*X* construct), leading to a significantly enhanced cell surface expression of the KAA*X* mutant when compared with WT (Fig. 1*E*). Secondly, we found that similar enhancement of cell surface expression of the β4-subunit was manifest in constructs in which a Myc tag (Myc_c_) was engineered at the very C terminus of the β4-subunit (Fig. 1, *D* and E). For example, surface expression of constructs that included both Myc_c_ and Myc_e_ tags was 5.5 ± 0.7-fold greater than constructs with the Myc_e_ tag alone. Combination of the KAA*X* mutation and Myc_c_ tag had no further effect on cell surface expression, suggesting that the C-terminal Myc_c_ tag masks the ER retention signal in the β4-subunit. Importantly, cell surface expression of the trafficking-competent β4-subunits (KAA*X* or Myc_c_ constructs) was dramatically reduced in palmitoylation-deficient β4-subunits with the C193A mutation (Fig. 1, *D* and E) with the palmitoylation-deficient subunits now predominantly localized to the ER (Fig. 1*F*). This suggests that palmitoylation of Cys-193 is important in controlling the exit of the β4-subunit from the ER. In accordance with trapping of the C193A β4-subunit mutant in the ER, the C193A mutation did not affect the mobility of the β4-subunit in SDS-PAGE (Fig. 1*B*), suggesting that core glycosylation of the β4-subunits, which occurs in the endoplasmic reticulum (16), was unaffected by the cysteine mutation. Furthermore, palmitoylation-dependent trafficking of the trafficking-competent β4-subunits was also observed upon overexpression in N2a neurons, revealing that this effect is not restricted to cell type. For example, surface expression of β4-subunits with the palmitoylation-deficient C193A mutation was expressed at 49.1 ± 3.3% of the WT palmitoylated β4-subunits in N2a neurons. In parallel, ER retention of the C193A β4-subunit mutant was increased when compared with the WT β4-subunits (Pearson's *R* was 0.72 ± 0.02 and 0.62 ± 0.04, respectively).

##### β4-Subunits Enhance Surface Expression of Pore-forming α-Subunits

**FIGURE 2.**
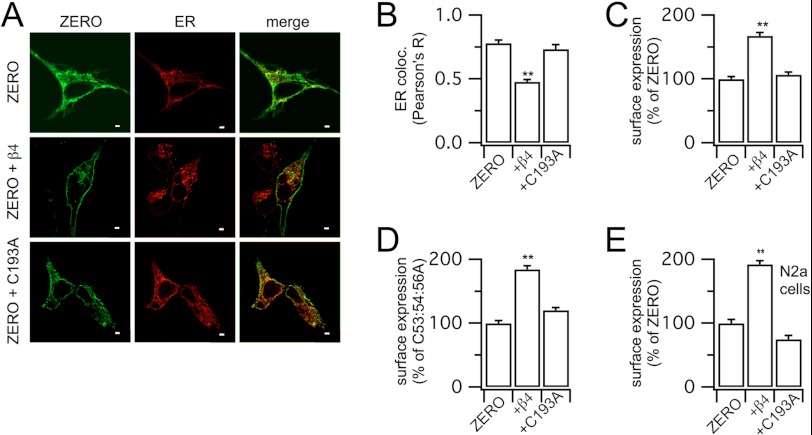
**β4-Subunit palmitoylation controls surface expression and ER retention of the pore-forming α-subunit ZERO variant of BK channels.** *A*, representative single confocal images of the ZERO variant of the pore-forming α-subunit of BK channels expressed in HEK293 cells in the absence and presence of the wild-type β4-subunit and the palmitoylation-deficient C193A subunit. Total α-subunit expression and co-labeling for the ER with merged images are shown. *Scale bars* are 2 μm. *B* and *C*, quantification of the effect of β4, or its C193A mutant, on ER co-localization (*ER coloc.*) (*B*) and cell surface expression of the ZERO α-subunit (*C*). *D*, β4, but not C193A, also increases cell surface expression of the ZERO α-subunit palmitoylation-deficient mutant C53:54:56A. *E*, recapitulation of cell surface enhancement of ZERO variant expression by β4 in the neuronal cell line N2a. For cell surface expression of ZERO α-subunit channel, protein was probed under nonpermeabilized (surface) and permeabilized (total) conditions, and the surface/total ratio was expressed as a percentage of the α-subunit in the absence of regulatory subunit as indicated. Data are means ± S.E., *N* > 7. **, *p* < 0.01 when compared with respective α-subunit expressed alone, ANOVA with post hoc Dunnett's test.

Previous studies have reported that β4-subunits may either down-regulate BK channel surface expression (15) or conversely enhance surface expression of the related pH-sensitive Kcnu1 (Slo3) pore-forming subunits (17). β4-Subunits assemble with the BK channel pore-forming α-subunits in the ER (16), and as depalmitoylated β4-subunits are retarded in the ER, we hypothesized that β4-subunits control the surface expression of α-subunits by restricting their exit from the ER. In initial studies, we used the ZERO variant of murine BK channels that encodes from the initiator methionine MDAL … and terminates in the C-terminal variant … REVEDEC (here also referred to as MDA-DEC, see Fig. 4*A*). We exploited a co-expression strategy in HEK293 cells and used quantitative immunofluorescence to determine the subcellular localization and trafficking of the ZERO variant in the presence and absence of the WT and C193A mutant β4-subunit. Expression of the ZERO variant alone leads to robust expression with a proportion of the channel localizing with the ER (Fig. 2, *A* and *B*) as well as at the plasma membrane (Fig. 2, *A* and *C*). Co-expression with WT β4-subunits resulted in a significant reduction of the ZERO channel variant co-localizing with the ER and subsequent increased expression at the cell surface (Fig. 2, *A–C*). This suggests that the WT β4-subunit facilitated ER export and trafficking of the ZERO variant to the cell surface. In contrast, expression of the C193A mutant of the β4-subunit had no significant effect on ER localization of the ZERO variant and did not result in an increased expression of the α-subunit at the cell surface (Fig. 2, *A–C*). Thus, the palmitoylation-deficient β4-subunit cannot facilitate ER export and surface expression of the ZERO variant.

Similar data were produced either using the extracellular Myc-tagged β4-subunit constructs and staining for surface expression using anti-Myc antibody or using untagged β4-subunits and staining with an β4-subunit antibody directed to an extracellular epitope. β4-Myc_e_ and β4-C193A-Myc_e_ increased ZERO surface expression by 174.7 ± 10.3 and 112.6 ± 8.2%, respectively, when compared with ZERO, whereas labeling with anti-β4 revealed an increase of 160.4 ± 7.9 and 103.5 ± 5.8% for WT β4 and β4-C193A, respectively. However, palmitoylation-deficient β4-subunits did not significantly suppress ZERO variant expression at the cell surface or enhance ZERO retention in the ER. This suggests that the β4-subunit normally acts to promote ZERO surface expression but that this is dependent upon β4-subunits being palmitoylated.

The ZERO variant itself is also palmitoylated at three cysteine residues within the intracellular S0-S1 loop (Cys-53, -54, and -56), and depalmitoylation of this site retards cell surface expression of the α-subunit (18, 19). We thus asked whether the palmitoylated β4-subunit could override the effect of depalmitoylation of the α-subunit and enhance its cell surface expression. Expression of the ZERO-C53:54:56A mutant, which cannot be palmitoylated, results in a significant decrease (reduced by 57.8 ± 6.3%) of cell surface expression when compared with the wild-type ZERO α-subunit alone. Co-expression of the ZERO-C53:54:56A mutant with WT β4-subunits resulted in rescue of surface expression of the depalmitoylated α-subunit to levels of the WT α-subunit (Fig. 2*D*). Again this was dependent on palmitoylation of the β4-subunits as the C193A β4-subunit mutant failed to rescue cell surface expression of the ZERO-C53:54:56A α-subunits (Fig. 2*D*). Thus, palmitoylated β4-subunits can override the inhibitory effects of ZERO α-subunit depalmitoylation on cell surface expression, suggesting that in cells that express β4-subunits, this mechanism may predominate.

β4-Subunits are predominantly, albeit not exclusively, expressed in many neurons and endocrine cells (6). We thus asked whether β4-subunit-mediated enhancement of α-subunit cell surface expression was recapitulated in neurons. To test this, we expressed the WT ZERO α-subunit alone or co-expressed with either WT β4-subunits or the C193A palmitoylation-deficient β4-subunits in murine N2a neurons. In agreement with the data in HEK293 cells, co-expression of the WT β4-subunits significantly enhanced surface expression of the ZERO variant, whereas the C193A β4-subunit mutant had no effect (Fig. 2*E*). This was again recapitulated with untagged β4-subunits as WT and palmitoylation-deficient C193A mutant β4-subunits increased ZERO surface expression to 219.7 ± 12.4 and 116.2 ± 4.3%, respectively, when compared with ZERO alone (100%) in N2a cells.

Importantly, these data reveal that in both HEK293 cells and N2a neurons, the ability of β4-subunits to enhance α-subunit surface expression is not dependent upon the ability of the β4-subunits *per se* to be able to traffic to the cell surface. Rather, the increased trafficking of ZERO is dependent upon the palmitoylation of the β4-subunit. In further support of this, although the ER retention-deficient β4-subunit mutant KAAK itself alone can traffic to the plasma membrane, in contrast to WT β4-subunits (Fig. 1), only β4-KAAK subunits that are palmitoylated enhance α-subunit surface expression. The ER retention-deficient β4-subunit mutant KAAK increased ZERO variant cell surface expression by 155.7 ± 7.6%, comparable with that observed with the WT β4-subunit, and this effect was abolished by the C193A mutation in the β4-KAAK mutant (surface expression was 101.6 ± 7.6% when compared with ZERO (100%) alone).

**FIGURE 3.**
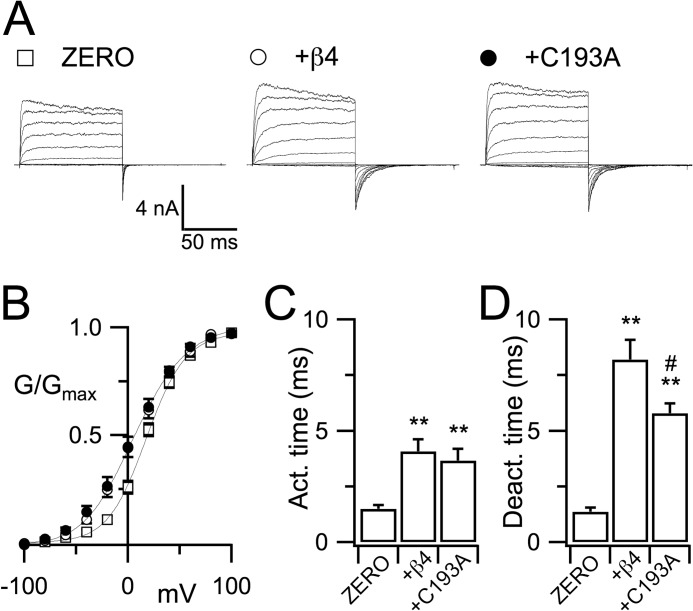
**β4-Subunit palmitoylation modifies channel deactivation kinetics.** *A*, representative macropatch recordings from isolated inside-out patches of HEK293 cells expressing the ZERO α-subunit variant (□) with and without WT β4 (○ or palmitoylation-deficient C193A β4-subunits (●) in the presence of 10 μm intracellular free calcium. *B*, corresponding normalized *G*/*V* relationships with Boltzmann fits determined from tail currents recorded as above. *C* and *D*, activation time (*Act. time*) constants determined at +60 mV (*C*) and deactivation time (*Deact. time*) constants determined at −60 mV (*D*). Data are means ± S.E., *n* = 10–17 per group. **, *p* < 0.01 when compared with ZERO expressed alone, #, *p* < 0.05 when compared with β4-subunit, ANOVA with post hoc Dunnett's test.

To investigate whether palmitoylation of the β4-subunit modified functional coupling of the β4-subunit with α-subunits at the cell surface, we undertook patch clamp electrophysiological analysis of co-expressed WT and C193A mutant β4-subunits with ZERO variants in HEK293 cells. Co-expression of WT β4-subunits resulted in a significant (*p* < 0.01, ANOVA) left shift (by 12.5 ± 2.9 mV) of the *V*_0.5 max_ determined from the conductance/voltage (*G*/*V*) relationship of tail currents recorded in 10 μm intracellular free calcium (Fig. 3, *A* and *B*). The C193A mutant displayed a similar left shift in *V*_0.5 max_ of 15.6 ± 3.6 mV (Fig. 3, *A* and *B*). The WT β4-subunit confers a significant slowing of both activation (Fig. 3*C*) and deactivation (Fig. 3*D*) kinetics of the ZERO variant. The C193A mutant displayed a similar slowing of activation kinetics (Fig. 3*C*). However, although deactivation kinetics were also significantly slowed when compared with ZERO alone, the deactivation time constant for the palmitoylation-deficient C193A mutant was significantly smaller than that observed with the WT β4-subunit (Fig. 3*D*). Taken together, although β4-subunit palmitoylation subtly modifies channel deactivation, these data support a predominant role for palmitoylation in controlling surface trafficking rather than the biophysical properties of the channel at the plasma membrane.

##### β4-Enhancement of α-Subunit Surface Expression Is Splice Variant-dependent

**FIGURE 4.**
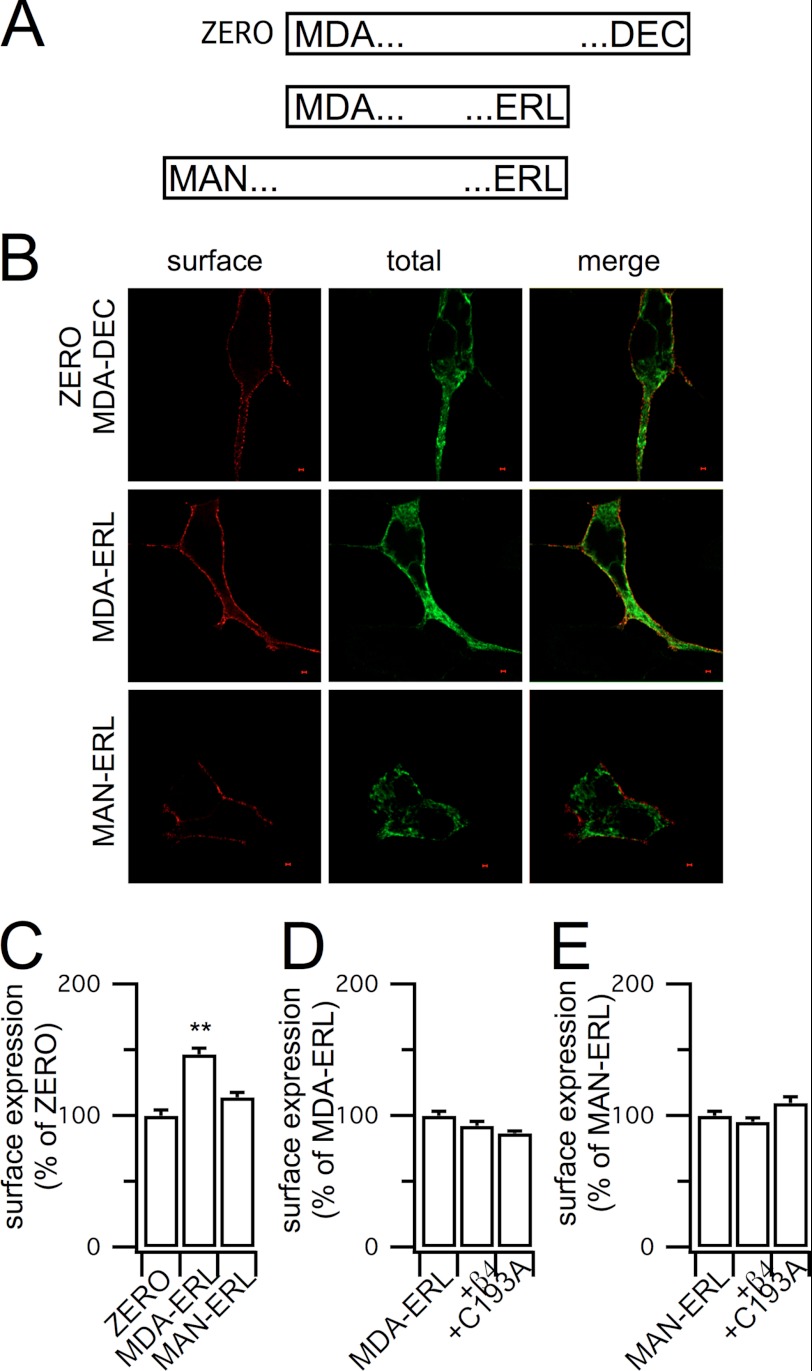
**β4-mediated enhancement of channel surface expression is α-subunit splice variant-dependent.** *A*, schematic of three distinct α-subunit splice variants that differ only in their very N or C termini, analyzed with the first and last 3 amino acids shown. The ZERO variant used in Fig. 2 is MDA-DEC with the variant with the same start methionine but shorter C terminus (MDA-ERL) and variant with upstream methionine with truncated C terminus (MAN-ERL) indicated. *B*, representative single confocal images of the surface (nonpermeabilized), total (permeabilized), and merged images of the three α-subunit splice variants. *Scale bars* are 2 μm. *C*, quantification of the surface expression of the three variants expressed as a percentage of ZERO (MDA-DEC) variant BK channels in HEK293. *D* and *E*, quantification of the effect of β4 or its C193A mutant on the MDA-ERL (*D*) and MAN-ERL (*E*) splice variants expressed as a percentage of the surface expression of the respective α-subunit alone. Data are means ± S.E., *N* > 5, *n* > 200. **, *p* < 0.01 when compared with respective α-subunit alone, ANOVA with post hoc Dunnett's test.

**FIGURE 5.**
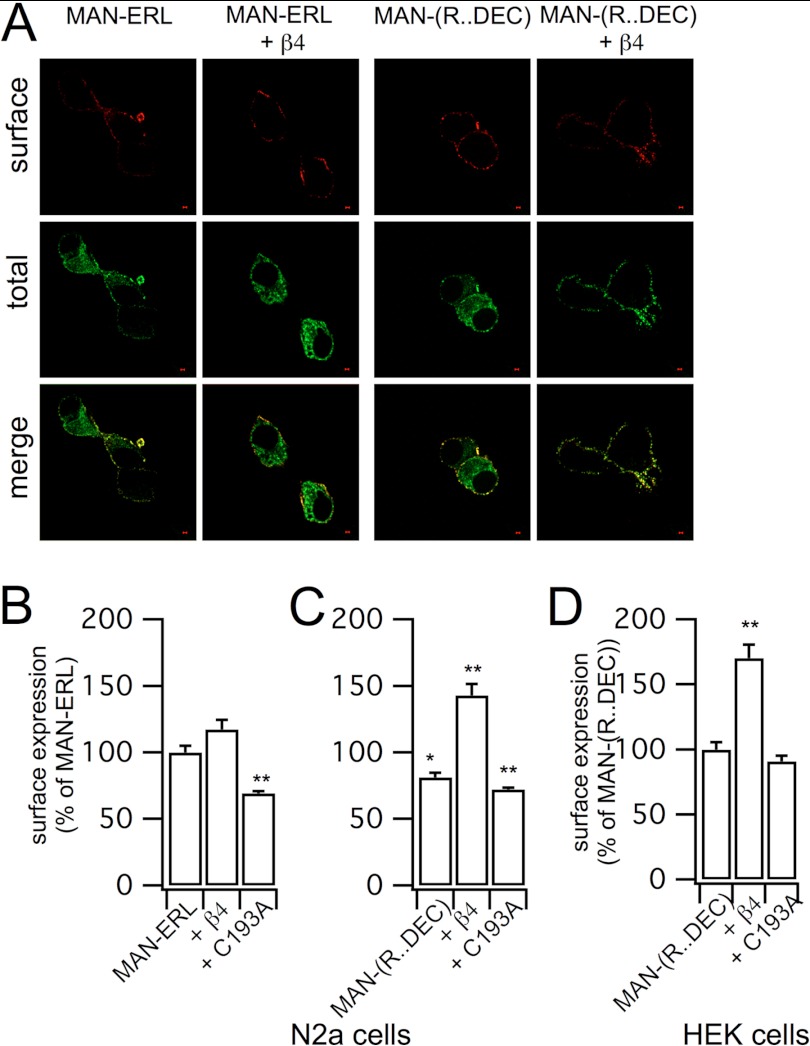
**The heptapeptide … REVEDEC is sufficient to confer β4-mediated enhancement of BK channel cell surface expression.** *A*, representative confocal images of the MAN-ERL α-subunit variant and the chimera in which the last 7 amino acids of MAN-ERL have been replaced by the heptapeptide REVEDEC (MAN-(R … DEC) expressed in N2a cells with or without the WT β4-subunit. *B* and *C*, quantification of surface expression of MAN-ERL (*B*) and MAN-(R … DEC) (*C*) in N2a cells expressed in the absence and presence of WT β4-subunit or the C193A mutant. Data are expressed as a percentage of MAN-ERL surface expression. *D*, cell surface expression of MAN-(R … DEC) in the presence or absence of WT β4-subunit or the C193A mutant expressed in HEK293 cells. Data are means ± S.E., *N* > 4, *n* > 96/group. *, *p* < 0.05, **, *p* < 0.01 when compared with MAN-ERL in *panels B* and *C* or MAN-(R … DEC) in *panel D* variant surface expression, ANOVA with post hoc Dunnett's test.

A recent study (15) reported that β4-subunits suppressed cell surface expression of BK channels in contrast to the data above. In contrast, β4-subunits have been reported to enhance surface expression of the related pH-sensitive pore-forming subunit encoded by Kcnu1 (17). In the former studies (15), BK channel α-subunit variants were used that differ in both the N termini and the C termini sequences when compared with the ZERO variant (MDA-DEC) used here. Taken together, these data suggested that β4-subunit-dependent trafficking may also be dependent upon the characteristics of the co-assembled α-subunit variant. To address this and to further understand the mechanism(s) by which β4-subunits promote ER exit and cell surface expression of the ZERO channels, we asked whether this effect was also mediated with other α-subunit splice variants. The very C terminus of the intracellular domain of BK channel α-subunits is subjected to alternative pre-mRNA splicing that has been reported to differentially control cell surface expression of the channel (20–23). In particular, α-subunits that contained the longest C-terminal splice variant that terminates in the heptapeptide sequence … REVEDEC sequence, as in our ZERO construct, display reduced cell surface expression when compared with α-subunit splice variants with shorter C termini that terminate in alternative sequences such as … QEERL and … VEMYR (20–23). Indeed, these studies demonstrated that swapping of the … VEDEC sequence to channels with the shorter C termini generated channel α-subunits that displayed a dominant negative motif for cell surface expression. Furthermore, transfection of cells with peptides encoding the … VEDEC sequence (20) or overexpression of a GFP fusion of the alternative spliced insert encoding the … VEDEC sequence (21) significantly increased cell surface expression of … VEDEC-expressing α-subunits. These data suggest that the … VEDEC peptide interacts with endogenous proteins to retard forward trafficking, although the mechanism and subcellular localization of trapped … VEDEC-containing α-subunits have not been defined (20). We thus hypothesized that the palmitoylated β4-subunit may mask interaction of … VEDEC with its endogenous target and thus promote α-subunit exit from the ER and enhance surface expression. We first verified whether swapping just the very C terminus of our ZERO α-subunits (which start with MDA … and terminate in … DEC, also referred to as MDA-DEC) with a shorter alternatively spliced C terminus increased surface expression of the α-subunit alone as reported previously (20, 21). To do this, we engineered in the C-terminal variant that terminates in the sequence … QEERL (Fig. 4*A*). This variant (MDA-ERL) when expressed alone showed a significantly increased cell surface expression when compared with the WT ZERO variant (*i.e.* MDA-DEC), as determined by quantitative immunofluorescence (Fig. 4, *B* and *C*). Co-expression of WT β4-subunits now had no effect on cell surface expression of the MDA-ERL variant (Fig. 4*D*). Similarly, co-expression with the C193A β4-subunit had no effect (Fig. 4*D*). These data thus suggest that the very C terminus of the pore-forming α-subunit is critical in determining the β4-mediated enhancement of cell surface expression. However, as surface expression of the MDA-ERL α-subunits alone was already significantly elevated when compared with WT ZERO, and in fact comparable with that observed upon co-expression of ZERO with WT β4-subunits, an alternative explanation could be that the surface expression of the MDA-ERL α-subunit is already maximal. To test for this possibility, we took advantage of another splice variant of the BK channel. This variant (MAN-ERL) has the same C terminus as for the MDA-ERL construct and only differs by having an extended extracellular N terminus, upstream of the MDAL … start site, with starting sequence MAN… . In our assays, this variant expresses at the cell surface with comparable levels when compared with the ZERO variant (*i.e.* MDA-DEC) α-subunits alone (Fig. 4, *B* and *C*). Co-expression with either WT or C193A mutant β4-subunits had no statistically significant effect on cell surface expression of the MAN-ERL α-subunits in HEK293 cells (Fig. 4*E*). However, in N2a neurons, the depalmitoylated (C193A) β4-subunits significantly reduced surface expression of the MAN-ERL α-subunit (Fig. 5*B*). Although the mechanism of this suppression remains to be resolved, this suggests that previous observations of β4-subunit-mediated down-regulation of CA3 hippocampal BK channels may represent conditions under which depalmitoylated β4-subunits assemble with distinct α-subunit splice variants (15). Taken together, these data suggest that the most C-terminal domain of ZERO is critical for the β4-mediated enhancement of cell surface expression of the ZERO (MDA-DEC) splice variant.

##### Palmitoylated β4-Subunits Mask a C-terminal Trafficking Motif in the Pore-forming ZERO α-Subunit Variant to Promote Cell Surface Expression

β4-Subunits only enhanced surface expression of α-subunit splice variants that included the extended C-terminal tail that terminates in … DEC. This strongly suggested that the mechanism of β4-mediated enhancement of cell surface expression is dependent upon motifs within this C-terminal splice insert. The final heptapeptide ( … REVEDEC) has been reported to reduce cell surface expression (20–23), and our data demonstrate that palmitoylated β4-subunits promote cell surface expression and facilitate ER export of α-subunits containing the … REVEDEC C terminus. We thus hypothesized that the … REVEDEC motif may act as a trafficking signal that may be masked upon expression with β4-subunits. If this was the case, we would predict that engineering the … REVEDEC sequence onto β4-subunit-insensitive α-subunits would result in depressed cell surface expression that could be rescued by WT, but not C193A mutant, β4-subunits. To determine whether the … REVEDEC sequence in fact behaved as a trafficking signal, we engineered the final 7 amino acids onto the C terminus of the MAN … ERL α-subunit variant to generate the chimera MAN-(REVEDEC). Fusion of … REVEDEC resulted in a significant reduction in cell surface expression of this α-subunit alone in both N2a neurons (Fig. 5, *A–C*) and HEK293 cells (Fig. 5*D*) with a concomitant increase in ER retention. Furthermore, co-expression with WT β4-subunits now rescued surface expression of the chimera toward levels observed with the MAN-ERL α-subunit and a significant (*p* < 0.01, ANOVA) reduction in co-localization of MAN-(REVEDEC) with the ER. Pearson's *R* for co-localization of MAN-(REVEDEC) with the ER was 0.88 ± 0.01, *n* = 6, and in the presence of WT β4-subunits, it was reduced to 0.76 ± 0.04, *n* = 8. Importantly, the β4-mediated increase in cell surface expression was dependent upon the palmitoylation status of the β4-subunits as the C193A mutant had no significant effect on cell surface expression of the chimera. These data strongly support a model in which the palmitoylated β4-subunit masks the C-terminal … REVEDEC trafficking motif to promote surface expression of α-subunit splice variants that include this sequence.

## DISCUSSION

Regulatory β4-subunits promote significant functional diversity in BK channels through modification of channel pharmacology, kinetics, surface trafficking, and complex effects on calcium/voltage sensitivity (6, 15, 16, 24, 25). Here we demonstrate that β4-subunits are regulated by the only *reversible* lipid post-translational modification of proteins, *S*-acylation (palmitoylation), in native tissues and heterologous expression systems. Importantly, *S*-acylation of β4 controls cell surface expression of the pore-forming α-subunit, an effect that is dependent upon alternative splicing of a trafficking signal ( … REVEDEC) in the very C terminus of the α-subunit. Using a chimera approach, we demonstrate that palmitoylated β4-subunits can specifically promote cell surface expression of α-subunits containing this motif. The data support a model in which β4-mediated enhancement of surface expression is mediated by β4-subunits masking the … REVEDEC trafficking signal as co-expression of β4-subunits enhanced α-subunit surface expression to a similar extent as removal of the … REVEDEC trafficking sequence. In such a model, why is β4-subunit palmitoylation a critical determinant? A plausible explanation is that palmitoylation may be important for the correct structural orientation of the β4-subunit with respect to the α-subunit to functionally mask the … REVEDEC signal. In this regard, the palmitoylated cysteine (Cys-193) is juxtaposed to the intracellular aspect of the second transmembrane domain of the β4-subunit. In other systems, juxta-transmembrane palmitoylation allows tilting of transmembrane domains, effectively shortening the transmembrane domain to both reduce hydrophobic mismatch (26), in particular at the thinner ER membrane (27), as well as induce conformational restraints on the peptide. Thus, the TM2 of depalmitoylated β4-subunits may display hydrophobic mismatch at the ER, reducing ER exit, and may have a conformation that is unfavorable for interaction with α-subunits. In this regard, disulfide cross-linking experiments (28) suggest that the extracellular aspect of TM2 of the β4-subunit is in close proximity to the S0 transmembrane domain of the α-subunit. Whether such a mechanism is important for control of trafficking that is dependent upon a motif ( … REVEDEC) at the very C terminus of the α-subunit remains to be determined.

*S*-Acylation of β4-subunits adds to the repertoire of post-translational mechanisms that can control BK channel function through the β4-subunit. For example, glycosylation of extracellular residues is important for determining the reduced efficacy of extracellular blockade by iberiotoxin (16), and phosphorylation of multiple intracellular residues is implicated in the control of functional interaction with α-subunits (29). Importantly, *S*-acylation provides a mechanism to control surface trafficking, and intriguingly, this effect is dependent upon the assembled α-subunit splice variant. A recent study (15) revealed that β4-subunits down-regulated surface expression of BK channel α-subunit variants with different C termini ( … KEMVYR), and other studies have shown that β4-subunits can enhance surface expression of Kcnu1 subunits (17). Together with our observation that surface expression of the MAN-ERL variant is suppressed only by *depalmitoylated* β4-subunits, this suggests that *S*-acylation of β4 may provide a specific regulatory signal to specifically control cell surface expression of BK channels assembled from different α-subunit splice variants containing the … REVEDEC sequence. Although the physiological consequence of such a mechanism remains to be determined, β4-subunits are important in a wide variety of physiological control systems ranging from dampening of excitability in the hippocampus (6) to regulation of potassium excretion from the kidney (5) and sensitivity of cells to alcohol (30) and neurosteroids (31). Furthermore, as *S*-acylation can be dynamically regulated, including by cell stress and diet (32), and β4 and α-subunit splice variant expression is spatially and temporally controlled (6, 8), this may provide a mechanism to allow fine tuning of specific physiological responses.

## Footnotes

2 The abbreviations used are:

ER: endoplasmic reticulum
ANOVA: analysis of variance
acyl-RAC: *S*-acylation by resin-assisted capture
HEDTA: *N*-(2-hydroxyethyl)ethylenediaminetriacetic acid
TM2: transmembrane domain 2.
